# Fellow-Workers and the Roll of Honour

**Published:** 1915-07-31

**Authors:** 


### ROUND THE WORLD.

[The Editor invites Early Information and Contributions Marked "Fellow-Workers."]

### Fellow-Workers and the Roll of Honour.

As noted in this Journal last week, Mr. Norman Lake and Captain C. H. Barber have made interesting reports to the *British Medical Journal* on the treatment of wounded limbs.

Mr. Norman Claudius Lake, who has been doing surgical work at a hospital for wounded at Nevers, France, reports very favourably on the plating of gunshot fractures; of all methods that he has tried, his opinion is that nothing gives such good results where a bone is badly smashed as holding together the fragments by suitable plates, this contention being supported by clinical reports and the evidence of *x*-ray photographs. Mr. Lake comes from Charing Cross Hospital, where he has held various appointments. An M.D., M.S., and B.Sc. of London University, he won the gold medal at the examinations for the first-mentioned degree; subsequently becoming an F.R.C.S. Eng., he was elected assistant surgeon to the Queen's Hospital for Children at Bethnal Green.

Captain Charles Harrison Barber, I.M.S., during the course of his work at No. 9 Indian General Hospital, has devised a new form of splinting for which he claims advantages in the treatment of gunshot wounds of the leg. Captain Barber is an Oxford and King's College Hospital man who qualified L.R.C.P., M.R.C.S. in 1902, previously winning a scholarship in medicine and surgery at his medical school. Before entering the Indian Medical Service, wherein he holds an appointment as specialist in operative surgery, he was house surgeon at King's College Hospital and house physician to the Victoria Hospital for Children at Chelsea.

Dr. Seymour Richard Gleed has left his practice at Finchley to take a commission in the R.A.M.C. A St. Thomas's Hospital man, he qualified in 1907, and held the posts of house surgeon to the Hospital for Women at Brighton, and senior house surgeon to the Scarborough Hospital, before settling down to private work.

Mr. Cecil Augustus Joll, assistant surgeon to the Royal Free Hospital, is taking up work as a member of the staff of the Euston Hospital for Officers (Endsleigh Palace Hotel). A distinguished representative of the Bristol School of Medicine, he won the gold medal and honours in medicine, surgery, and forensic medicine at the M.B., B.S. Lond. in 1909, as well as numerous collegiate scholarships and prizes. Mr. Joll, who did clinical work at University College and the London Hospital after leaving Bristol, is possessed of numerous academic distinctions; in addition to being a London graduate in medicine and surgery, he is a B.Sc. Lond., M.S. Lond., F.R.C.S. Eng., and M.B., Ch.M. Bristol.

Another member of the staff of the institution referred to in the last paragraph is Mr. Hunter Tod, one of our leading authorities on diseases of the ear and throat. Mr. Hunter Tod, who is surgeon to the ear, nose, and throat department of the London Hospital, and lecturer on his special subjects in the medical school attached thereto, is an M.D. Cantab. and F.R.C.S. Eng. Examiner in otology to the Royal Army Medical College, he was at one time surgeon to the throat and ear department of the Paddington Green Children's Hospital, and senior resident medical officer at the Golden Square Throat Hospital.

Major David Robert Taylor, R.A.M.C. (T.F.), who was recently killed in the Dardanelles campaign, had been practising at St. Helens, Ayton, in East Berwickshire, for some twenty years when the war broke out; he mobilised as a medical officer attached to the King's Own Scottish Borderers (4th Battalion). An Edinburgh student, Major Taylor qualified by taking the "triple" Scottish diploma of Edinburgh and Glasgow in 1893, and soon after settled in private practice. Always keenly interested in the Volunteer movement, he obtained a commission not long after going to reside at St. Helens. His appointments included those of visiting physician to the Millerton Hospital for Infectious Diseases and medical officer for the Post Office.

Dr. George Basil Price, who has accepted a temporary commission in the R.A.M.C. for work with one of the chief clearing hospitals, is well known in connection with the London Missionary Society. A "Bart's." man, he qualified at the Conjoint Board's examinations, and subsequently became an M.D., B.S. Lond., M.R.C.P. Lond., D.P.H.

Mr. Rhys Evans, who was recently killed, left Guy's Hospital about three years ago on taking a dental diploma. As a student of the dental school attached to the hospital he had distinguished himself as a good sportsman, and for some years regularly defended the goal for the Guy's First Eleven Association Football Team.

The two wards in the Royal Victoria Hospital, Edinburgh, set apart for the treatment of men suffering from nervous exhaustion and shock are under the direction of three Commissioners of the General Board of Control for Scotland—namely, Dr. John Carswell, Dr. John Macpherson, and Dr. Hamilton Marr.

Dr. John Carswell, J.P., is a diplomate of Edinburgh and Glasgow, and was educated at the well-known Anderson's College in the last-named city. Taking up the problems of mental disorder as his special study, he soon made for himself an authoritative position, eventually obtaining his present important post of Commissioner for Scotland. Dr. Carswell's past appointments include those of certifying physician in lunacy for Glasgow parish, medical officer for mentally defective children under the Glasgow School Board, physician to the mental department of the Eastern District Hospital in Glasgow, and lecturer on mental diseases at Anderson's College.

Dr. John Macpherson is president of the Medico-Psychological Association for the current year. Qualifying at Edinburgh University in 1882, he became an F.R.C.P. Edin. in 1891, and won a gold medal at the M.D. Edin. five years later. Dr. Macpherson has had very great experience of insanity, both from the clinical and administrative points of view, having been medical superintendent of the Stirling District Asylum and senior assistant physician under the late Sir Thomas Clouston at the Royal Asylum, Morningside, Edinburgh. He was formerly lecturer on mental diseases at the Edinburgh Royal School of Medicine, and some years ago wrote a book on "Mental Affections."

